# Cecal Endometriosis Presenting as Hematochezia in a Postmenopausal Female

**DOI:** 10.7759/cureus.33886

**Published:** 2023-01-17

**Authors:** Roland J Quintana-Rapatalo, Davong D Phrathep, Ivanna Ward, Kevin D Healey, Stefan Anthony, Michael Herman

**Affiliations:** 1 College of Medicine, Florida International University, Herbert Wertheim College of Medicine, Miami, USA; 2 College of Osteopathic Medicine, Lake Erie College of Osteopathic Medicine, Bradenton, USA; 3 College of Osteopathic Medicine, Philadelphia College of Osteopathic Medicine, Philadelphia, USA; 4 Gastroenterology, Borland Groover, Jacksonville, USA

**Keywords:** cyclical abdominal pain, postmenopausal female, cecal endometriosis, endometriosis, hematochezia

## Abstract

Our report highlights the diagnosis of cecal endometriosis as a unique cause of hematochezia in a postmenopausal female. Cecal endometriosis manifesting as intermittent hematochezia and abdominal pain is uncommon but requires prompt clinical diagnosis and management. We report a case of cecal endometriosis causing hematochezia and subsequent syncope, which prompted the patient’s admission to the emergency department. In our patient, a diagnosis of cecal endometriosis was made after a colonoscopy, with multiple biopsies confirming the presence of endometrial tissue embedded in the cecum. We aim to bring awareness of cecal endometriosis presenting as hematochezia in a postmenopausal woman with a history of abdominal pain. This case highlights intestinal endometriosis as a differential diagnosis to be considered in women, regardless of age, with intermittent hematochezia and abdominal pain.

## Introduction

Endometriosis is a condition where endometrial tissue is found outside of the uterus [1]. It affects 6-10% of women worldwide, who are commonly of reproductive age [2]. Common symptoms of endometriosis include menorrhagia, dysmenorrhea, and dyspareunia although patients can remain asymptomatic [1]. One of the most common sites of extragenital implantation is along the bowel and bowel-related implants can manifest as dyschezia or hematochezia [3]. Endometriosis causes right iliac fossa pain, especially during menstrual periods because the endometrial-like tissue acts similar to the endometrium, where it thickens, breaks down, and bleeds with each menstrual cycle [3]. If endometrial tissue involves the bowels, it can cause intermittent abdominal pain, especially during menstrual periods [4]. In recent literature, endometriosis has been documented in postmenopausal females and has the potential to implant in the bowels. When endometrial tissue is implanted in the bowels, it usually occurs in the rectosigmoid and less frequently occurs in the cecum [4]. Cecal endometriosis can present as acute appendicitis, intussusception, volvulus, chronic abdominal/pelvic pain, or bowel obstruction [4]. However, in this report, we present a new clinical presentation of cecal endometriosis presenting as hematochezia in a postmenopausal female.

## Case presentation

A 51-year-old post-menopausal female reports to the emergency department for syncope and gastrointestinal bleeding. The patient’s husband immediately notified emergency medical services (EMS) when the patient was found on the floor with blood surrounding her. EMS described the patient’s blood as dark red. The patient reported two episodes of diarrhea the night of the incident but did not recall her syncopal episode. She reported that the onset of the syncope and gastrointestinal bleeding only happened one day prior to the incident and that the course and duration of her symptoms were intermittent. She had been suffering from intermittent hematochezia and reported severe abdominal pain during these episodes for the past month. Her medical history revealed no medical conditions related to her complaints. There were no recent changes in bowel habits or weight loss. She had no family history of colorectal cancer or inflammatory bowel disease. She denied tobacco and alcohol use. The patient had a previous colonoscopy a month prior to the episode, which revealed benign findings.

On admission, the patient was alert and oriented to person, place, time, and event. The patient appeared slightly pale and showed signs of apparent distress. Vital signs upon admission showed a blood pressure of 117/65 mmHg, pulse of 67 per minute, temperature of 97.88 degrees Fahrenheit, respiratory rate of 18 per minute, and oxygen saturation of 99%. The patient denied nausea, vomiting, hematemesis, hemoptysis, fever, chills, and urinary abnormalities. A physical exam revealed a tender abdomen in the right lower quadrant with mild pain with palpation. Labs revealed low hemoglobin at 9.9 g/dL and low hematocrit at 29.4%. The patient’s partial thromboplastin time was low at 22.8 seconds and glucose levels were high at 119 mg/dL. The remainder of the patient’s lab values were stable. A pneumatic compression device was applied for deep vein thrombosis prevention.

**Figure 1:**
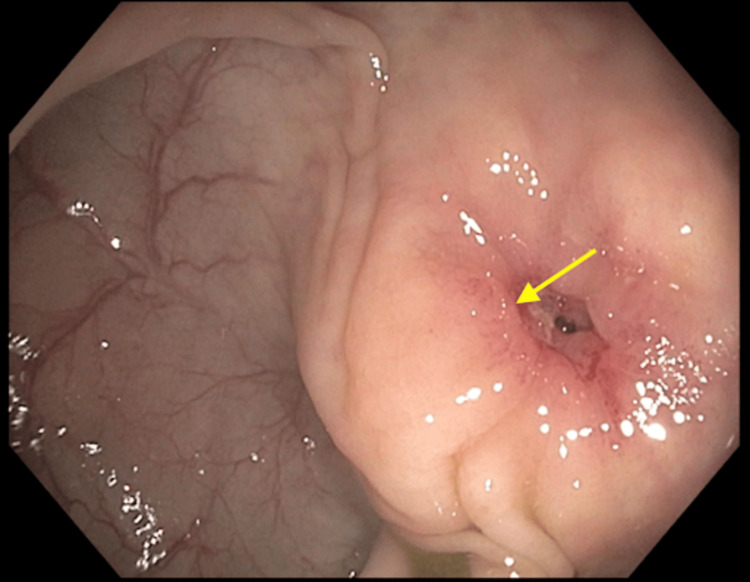
Colonoscopy image of an erythematous nodule embedded in the cecum

The patient underwent CT angiography of the abdomen and pelvis, which revealed no evidence of an active gastrointestinal bleed. There was no evidence of significant stenosis, dissection, or aneurysm. The patient was advised about her current situation, and she was discharged with scheduled follow-up visits with gastroenterology. A follow-up colonoscopy revealed an erythematous nodule embedded in the cecum (Figure 1).

Multiple biopsies were taken from the cecum. The pathologic examination showed ectopic endometrial glands in the cecum. There was no microscopic evidence of any other pathologies. Given the absence of other possible etiologies, we attribute her hematochezia to cecal endometriosis. The patient was recommended to follow up with an outpatient gynecologist for a consultation.

## Discussion

Endometriosis is defined as an ectopic proliferation of endometrial tissue outside the uterine cavity [5]. It is fairly common in women of childbearing age. Bowel involvement in endometriosis is uncommon and usually localized in the rectosigmoid and less frequently in the cecum. Among instances of bowel endometriosis, the cecum, along with the appendix and ileocecal junction, were found to be the third most common site of implantation [5]. To date, there have been two documented findings of cecal endometriosis in a postmenopausal female but none that have presented with hematochezia [6,7]. The prevalence of endometriosis in postmenopausal females is reported to be between 2% and 5% [8]. In the hypoestrogenic state of menopause, symptoms of endometriosis are expected to decrease due to the atrophy of endometriotic lesions. However, there have been descriptions of recurrent endometriotic lesions presenting in a postmenopausal female [9]. Instances of recurrence are theorized to be due to exogenous estrogen use or peripherally produced estrogen causing reactivation of the lesions. De novo mutations have also been previously described. As the patient was no longer menstruating, metaplasia of the parietal peritoneum is more likely an explanation.

The underlying pathophysiology of this patient’s condition is likely multifactorial and may be explained by historical etiological hypotheses of endometriosis, which are mostly found to be in women of reproductive age. Consistent with the retrograde menstruation hypothesis, a clockwise flow of peritoneal fluid containing refluxed endometrium can deposit on the cecum [10]. Additionally, the parietal peritoneum could undergo coelomic metaplasia to endometrial-like cells especially due to the close proximity of the bowel to the peritoneum or right abdominal side wall [10]. Regardless of the means of deposition, the infiltrating endometrial tissue then can grow into the intestines, which is what ultimately caused blood to begin presenting within the stool, which has been a notable finding in earlier reports [11].

The diagnosis of endometriosis, especially cecal endometriosis in an emergent setting, is difficult clinically [4]. Current literature comments on cecal endometriosis presenting as acute appendicitis, intussusception, Crohn's disease, volvulus, chronic abdominal/pelvic pain, tubo-ovarian abscess, cecal diverticulitis, ileocecal tuberculosis, or bowel obstruction [4]. Our case highlights the need for clinical suspicion of endometriosis as a differential diagnosis to be considered in women of any age who are presenting with intermittent hematochezia and abdominal pain. As the patient had cyclical abdominal pain, it also highlights the importance of screening questions related to menstrual irregularities while obtaining the history and physical. Our case highlights cecal endometriosis as a cause of intermittent hematochezia and severe abdominal pain in a postmenopausal female.

Typically, surgical removal of the endometrium tissue via laparoscopy or laparotomy from the bowels is the most common treatment. Depending on the location, severity, and size of bowel involvement, patients can undergo segmental bowel resection, rectal shaving, or disc resection for the treatment of bowel endometriosis. A right hemicolectomy is a procedure that can be performed by laparotomy and has been used in the current literature for the treatment of cecal endometriosis [4]. For cecal endometriosis, surgery may be considered to avoid complications like additional bleeding, perforation, bowel obstruction, and malignant tumor formation [4]. It is important to consult with a gynecologist to determine the appropriate surgery that needs to be performed. Our patient was consulted by gastroenterology for the presentation of hematochezia and then was referred to a gynecologist for consultation and treatment recommendations.

## Conclusions

Endometriosis is often a painful disorder in which endometrial tissue grows outside of the uterus. Endometriosis most commonly involves the ovaries, fallopian tubes, and pelvis lining. Rarely, endometrial tissue may be found beyond areas where the pelvic organs reside. Bowel involvement in endometriosis is uncommon, but when it occurs, it is usually localized to the rectosigmoid and less frequently in the cecum. This report is that of a 51-year-old female who presented with a history of intermittent hematochezia and severe abdominal pain for a month. Our case highlights cecal endometriosis as a differential diagnosis to be considered in women, regardless of age, with intermittent hematochezia. Current literature comments on cecal endometriosis presenting as acute appendicitis, intussusception, volvulus, chronic abdominal/pelvic pain, or bowel obstruction. However, our report identifies an additional clinical presentation of cecal endometriosis presenting as hematochezia. A high index of suspicion is required for diagnosis, especially if the woman has a history of cyclical abdominal pain. We urge further research to be completed to identify additional unique clinical presentations of cecal endometriosis.
